# Carbon range verification with 718 keV Compton imaging

**DOI:** 10.1038/s41598-021-00949-5

**Published:** 2021-11-04

**Authors:** Raj Kumar Parajuli, Makoto Sakai, Kazuo Arakawa, Yoshiki Kubota, Nobuteru Kubo, Mutsumi Tashiro

**Affiliations:** 1 Department of Molecular Imaging and Theranostics, National Institutes for Quantum Science and Technology, 4-9-1 Anagawa, Inage, Chiba, 263-8555 Japan; 2 Gunma University Heavy Ion Medical Center, Gunma University, 3-39-22 Showa-machi, Maebashi, Gunma Japan; 3 B Dot Medical Inc., 5-10-10 Harue-cho, Edogawa-ku, Tokyo, Japan; 4 Department of Radiation Oncology, Graduate School of Medicine, Gunma University, 3-39-22 Showa-machi, Maebashi, Gunma Japan

**Keywords:** Applied physics, Particle physics

## Abstract

Carbon ion radiotherapy is a sophisticated radiation treatment modality because of its superiority in achieving precise dosage distribution and high biological effectiveness. However, there exist beam range uncertainties that affect treatment efficiency. This problem can be resolved if the clinical beam could be monitored precisely in real-time, such as by imaging the prompt gamma emission from the target. In this study, we performed real-time detection and imaging of 718 keV prompt gamma emissions using a Si/CdTe Compton camera. We conducted experiments on graphite phantoms using clinical carbon ion beams of 290 MeV/u energy. Compton images were reconstructed using simple back-projection methods from the energy events of 718 keV prompt gamma emissions. The peak intensity position in reconstructed 718 keV prompt gamma images was few millimeters below the Bragg peak position. Moreover, the dual- and triple-energy window images for all positions of phantoms were not affected by scattered gammas, and their peak intensity positions were approximately similar to those observed in the reconstructed 718 keV prompt gamma images. In conclusion, the findings of the current study demonstrate the feasibility of using our Compton camera for real-time beam monitoring of carbon ion beams under clinical beam intensity.

## Introduction

Carbon ion radiotherapy (CIRT) is a widely accepted modality for the treatment of deeply seated tumors because of its high-dose localization around the Bragg peak (BP)^1^ and its biological effectiveness^2–5^. However, these unique properties make CIRT more sensitive to beam range uncertainties^6–8^. Unintended irradiation causes unnecessary damage to healthy tissues and reduces tumor doses^9,10^. One of the methods used to reduce uncertainties is the real-time monitoring of beams based on secondary radiation measurement. In this regard, a positron emission tomography (PET) system is one of the potential systems^11–16^. However, high-energy positrons and annihilation gamma rays emitted via pair production are less related to the range^17^. On the contrary, C-11, N-13, and O-15 are affected by the metabolic washout effect because of their long half-lives^18^. Owing to these difficulties and large detector size, in-beam PET has limited detection efficiency as a real-time beam monitoring system^19–21^.

Apart from the positron annihilation products, the relaxation of the excited nuclei also produces secondary photons, emitting gamma rays of a wide energy range^22^ from hundreds of keV to 10 MeV. Because prompt gamma rays are usually generated within nanoseconds, they are not prone to washout effects^23^. Prompt gamma-ray imaging at lower energies below 1 MeV, such as 718 keV, could be efficient in terms of washout effects, gamma detection efficiency, detector size, portability, and economic investments^24^ than do high-energy gamma rays. The projectile ^12^C ions collide with the target nuclei and produce ^10^B* (0.7 ns half-life) via the nuclear spallation or β+ decay of ^10^C^25,26^, and ^10^B* generates 718 keV gamma rays through the relaxation. Therefore, the distribution of the generated 718 keV gamma rays would correlate with the carbon beam range, although the exact distribution of the amount of gamma emissions within the track remains unclear. In addition, 718 keV prompt gamma rays are also expected to be an objective measurement for whole gamma imaging^27^.

**Figure 1:**
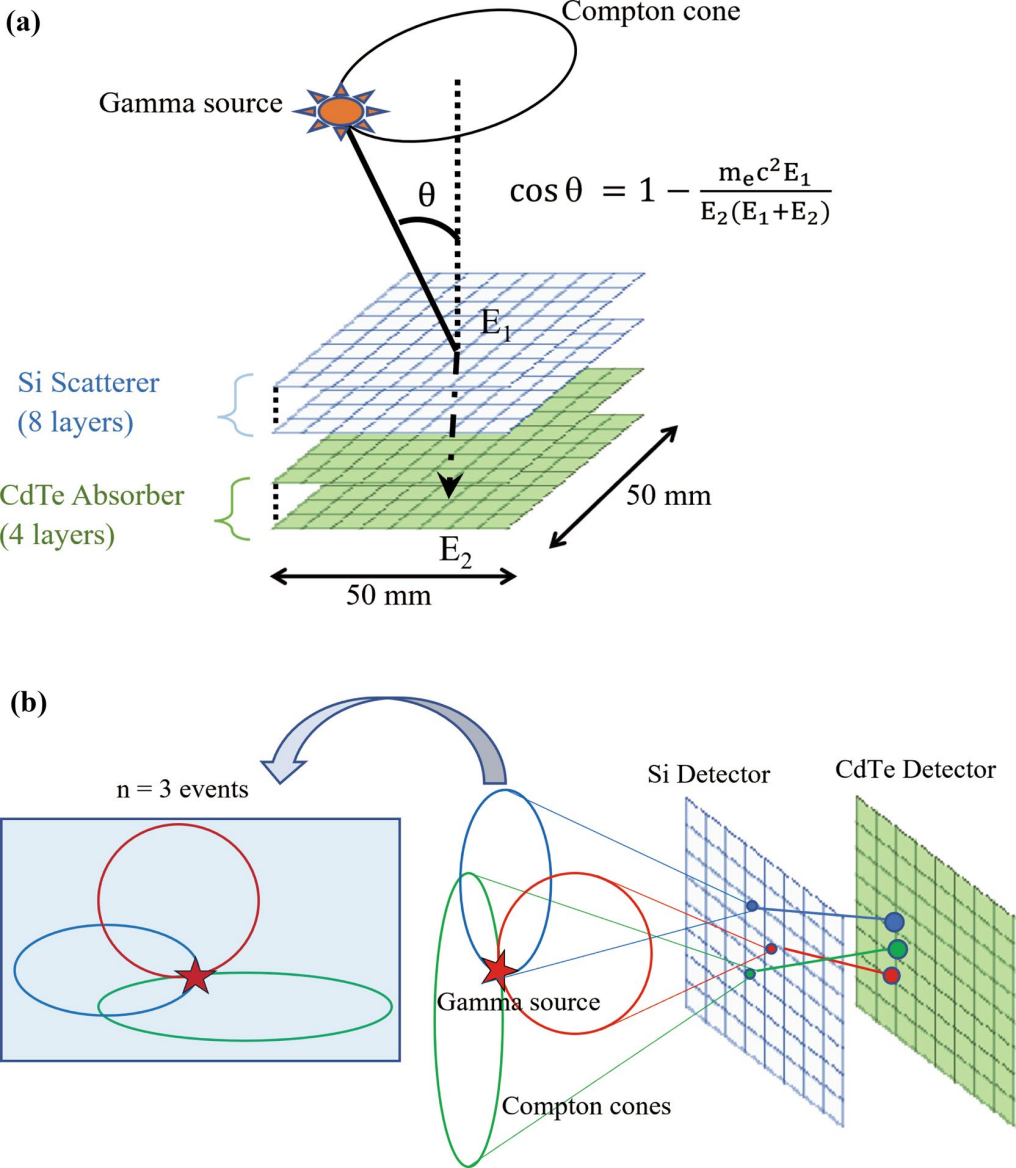
Schematic illustration of Compton camera. (**a**) Alignment of semiconductors within our Compton camera. (**b**) Gamma source detection with increased number of overlapping events.

Although the Compton camera was originally introduced for astronomy^28,29^, it shows potential for application in particle therapy for beam range monitoring^30–32^. The beam monitoring ability of our Compton camera for real-time annihilation gamma imaging has been successfully demonstrated^33,34^. The main advantages of a Compton camera are its compact size and its wide energy range (100 keV to several MeV)^35,36^ based purely on Compton kinematics without the use of mechanical collimators^37^. A fundamental Compton camera consists of two types of sub-detectors called scatterers and absorbers (Fig. 1a). For an individual gamma emission, Compton scattering occurs in the scatterer, and photoabsorption occurs in the absorber. We call these scattering and absorption events together with a Compton event. A cone is formed by the scattered angle θ in the scatterer. The cones are accumulated together to determine the gamma source (Fig. 1b). The scattered angle θ is calculated as follows:

1 $$cos\theta = 1 - (m_{e} c^{2} E_{1} )/(E_{2} (E_{1} + E_{2} ))$$

where m_e_ c^2^ is the mass energy of an electron, E_1_ is the energy of the recoiled electron in the scatterer, and E_2_ is the energy deposited in the absorber.

Currently, we are developing a Si/CdTe-based Compton camera for medical purposes and exploring its applications^33,34,38–43^. Si detectors have a smaller Doppler broadening than other semiconductors^44^, and the employed CdTe detector comprises high-resolution Schottky diodes^45^. Therefore, the energy resolution of the Si/CdTe camera is high and leads to higher angular resolution than that of other commercial gamma cameras. In this study, owing to the superior features of our Si/CdTe Compton camera and the advantage of measuring the on-beam prompt gamma emissions, we performed real-time measurement of 718 keV prompt gamma rays emitted by a therapeutic carbon ion beam. This study provides valuable information regarding real-time beam range monitoring since, to date, there have been no detailed reports of beam range estimation method using 718 keV gamma rays, neither with the Compton camera nor with conventional gamma cameras.

## Results

### On-beam range verifications using prompt gamma emission

**Figure 2:**
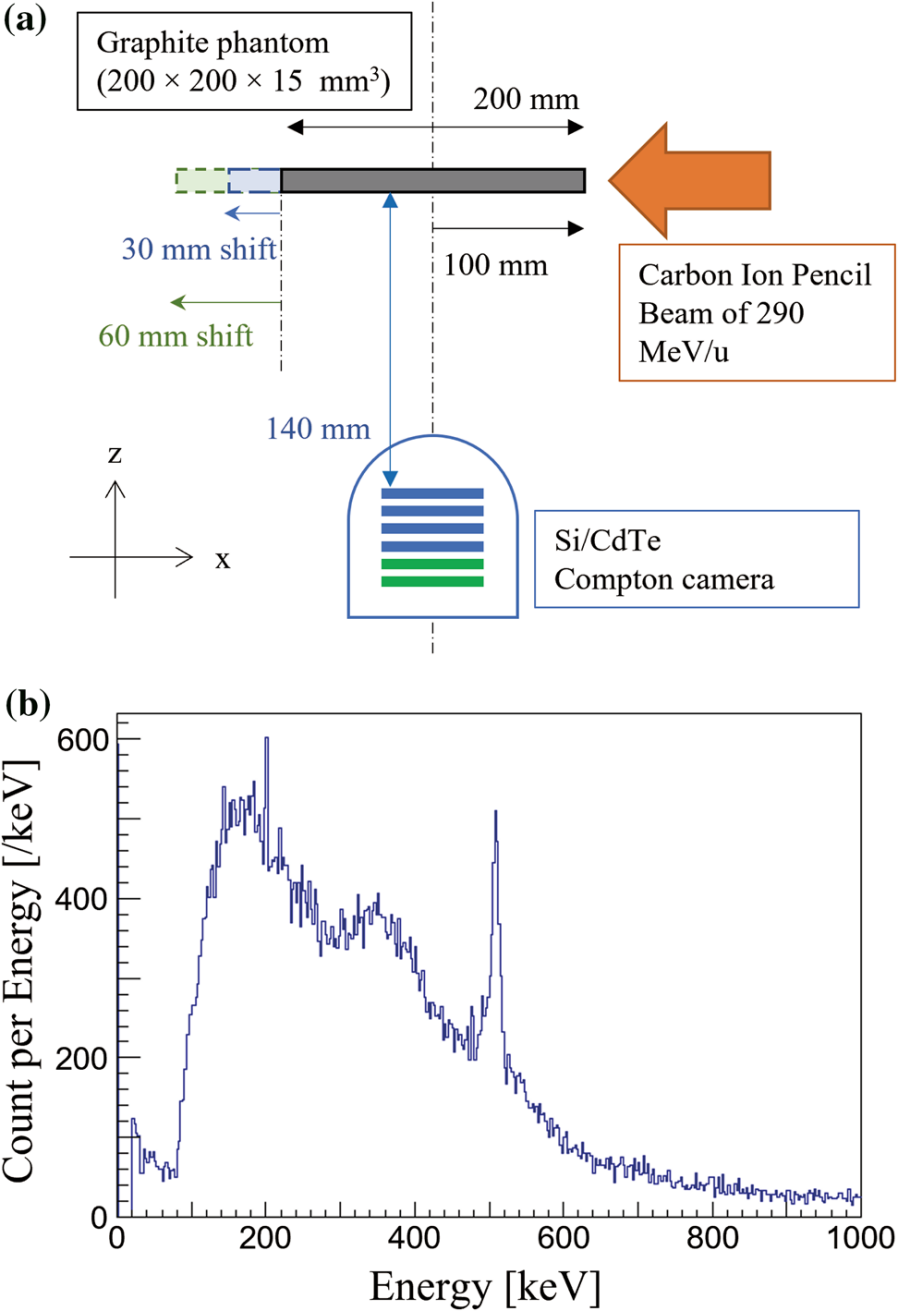
(**a**) Schematic illustration of the experimental setup. Graphite phantoms at the origin and those shifted by 30 mm and 60 mm were irradiated with a clinical carbon ion pencil beam of 290 MeV/u energy. (**b**) Energy spectrum measured by Compton camera under the beam-on condition in a graphite phantom placed at the origin (0 mm).

**Figure 3:**
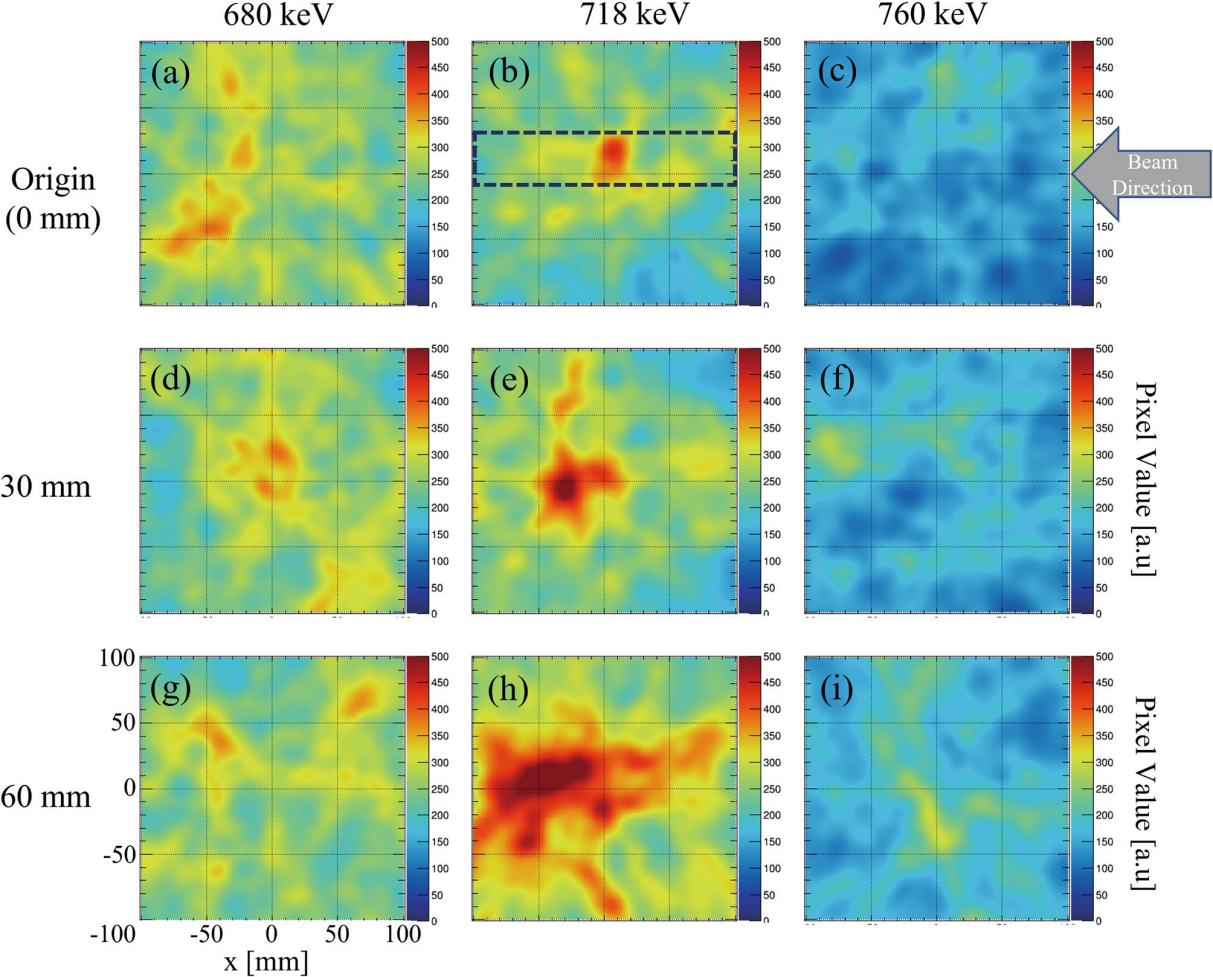
(**a**), (**d**), and (**g**) Reconstructed back-projection Compton images of 680 keV; (**b**), (**e**), and (**h**) 718 keV; and (**c**), (**f**), and (**i**) 760 keV acquired using the graphite phantoms positioned at origin (0 mm), 30 mm, and 60 mm, respectively. The dotted line in (**b**) indicates the projection range used for calculation of the x-projection profiles of 718 keV Compton images (**b**), (**e**), and (**h**). The arrow in (**c**) shows the direction and location of the beam entrance in graphite phantom.

The 718 keV prompt gamma emissions from graphite phantom placed at different positions were measured using the Compton camera for the experimental setup illustrated in Fig. 2a. Figure 2b shows the energy spectrum measured from the graphite phantom placed at the origin using our Compton camera. The numbers of selected Compton events within the photo-peak window of 718 keV during the 10-min detection were 263, 265, and 336 from the phantom at origin (0 mm), 30 mm, and 60 mm, respectively. The detection ratio of a Compton event per projectile was approximately 5 × 10^–10^. The live time ratio evaluated for the phantom placed at 0 mm position was 8%. Figure 3 shows the reconstructed back-projection images of 680 keV (Fig. 3a,d,g), 718 keV (Fig. 3b,e,h), and 760 keV (Fig. 3c,f,i) for the phantoms positioned at 0 mm, 30 mm, and 60 mm, respectively. The arrow in Fig. 3c shows the direction and location of the beam entrance position in all the experiments. Although a distinct energy peak of 718 keV was not visible in the energy spectrum (Fig. 2b), peak intensity positions of 718 keV prompt gamma emissions could be successfully observed in the reconstructed Compton images. This was because the background component of the signal detected by the Compton camera was large; thus, the 718 keV signal was buried in the noise in the energy spectrum. However, the noise detected by the Compton camera from different directions had a lesser effect on the reconstructed Compton image. Because the data measured by the Compton camera is a list of all data of gamma rays generated from all the space (list-mode method) that includes the main signal and the noise from a wide background region. Thus, the pixel values related to the noise are diluted, however, the pixel values reconstructed by the signal are concentrated in the particle beam trajectory. Therefore, even though the signal-to-noise ratio is poor in the energy spectrum, the reconstructed image shows carbon beam trails. The reconstructed Compton images of 680 keV and 760 keV at respective phantom positions did not appear to have peak intensity gamma emissions (Fig. 3a,c,d,f,g,i) that trace the BP position.

**Figure 4:**
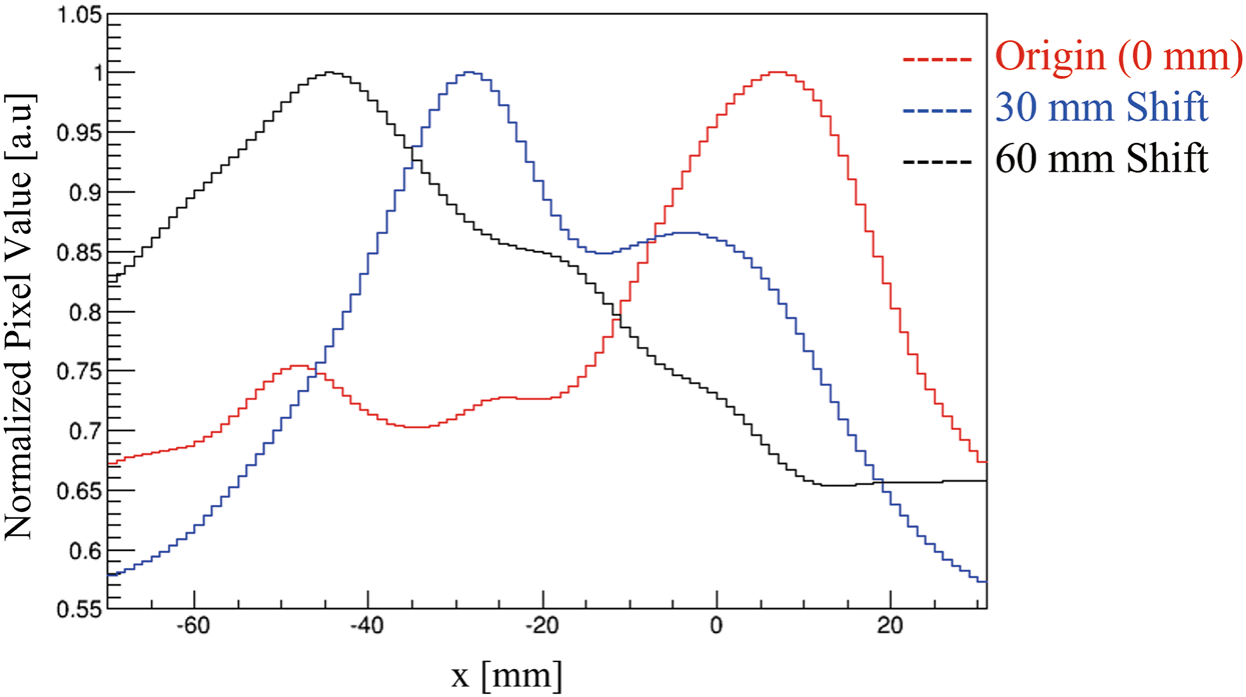
Normalized x-projection profile of the reconstructed Compton images of 718 keV illustrated in Figure 3, acquired using the graphite phantoms positioned at: 0 mm, red line; 30 mm, blue line; and 60 mm, black line. The x-projection profile was calculated within the projection range indicated by the dotted line in Fig. 3b.

The peak intensity position of the prompt gamma emissions was evaluated by plotting a y-direction projection profile on the reconstructed Compton images (Fig. 4) at the origin (0 mm, red line), 30 mm shift (blue line), and 60 mm shift (black line) positions. Y-axis values were normalized using the maximum values. The projection range of 20 mm was approximately twice the diameter of beam size, and a Gaussian function was fit within 30 mm in the x-direction. The dotted rectangular line in Fig. 3b indicates the projection range used for evaluating the x-projection profile of Fig. 3b,e,h to plot their respective x-projection profile in Fig. 4. Table 1 shows the numerical value of BP position calculated using Monte Carlo simulation, peak intensity position in 718 keV Compton image estimated by Gaussian fitting on the x-projection profile, the difference of their peak intensity position, and peak shift from the origin. The estimated experimental values may have been affected by some random and systematic errors such as those caused by a manual shifting of the phantom and projection evaluation on the reconstructed image using the appropriate Gaussian fitting method and projection range.

**Table 1:** Numerical table illustrating Bragg peak (BP) position evaluated using Monte Carlo simulation, position of peak intensity of 718 keV Compton image, difference of 718 keV peak intensity from BP positions, and the shift of 718 keV peak intensity positions from the origin for the graphite phantom positioned at 30 mm and 60 mm.

Graphite phantom positions	0 mm (origin)	30 mm	60 mm
Bragg peak position evaluated from simulation (mm)	98.4	128.4	158.4
Peak intensity positions in 718 keV Compton image (mm)	94.4	128.2	145.1
Difference of 718 keV peak intensity from BP positions (mm)	− 4.0	− 0.2	− 13.3
Shift of 718 keV peak intensity positions from the origin (mm)		33	50

### Dual- and triple-energy window imaging

**Figure 5:**
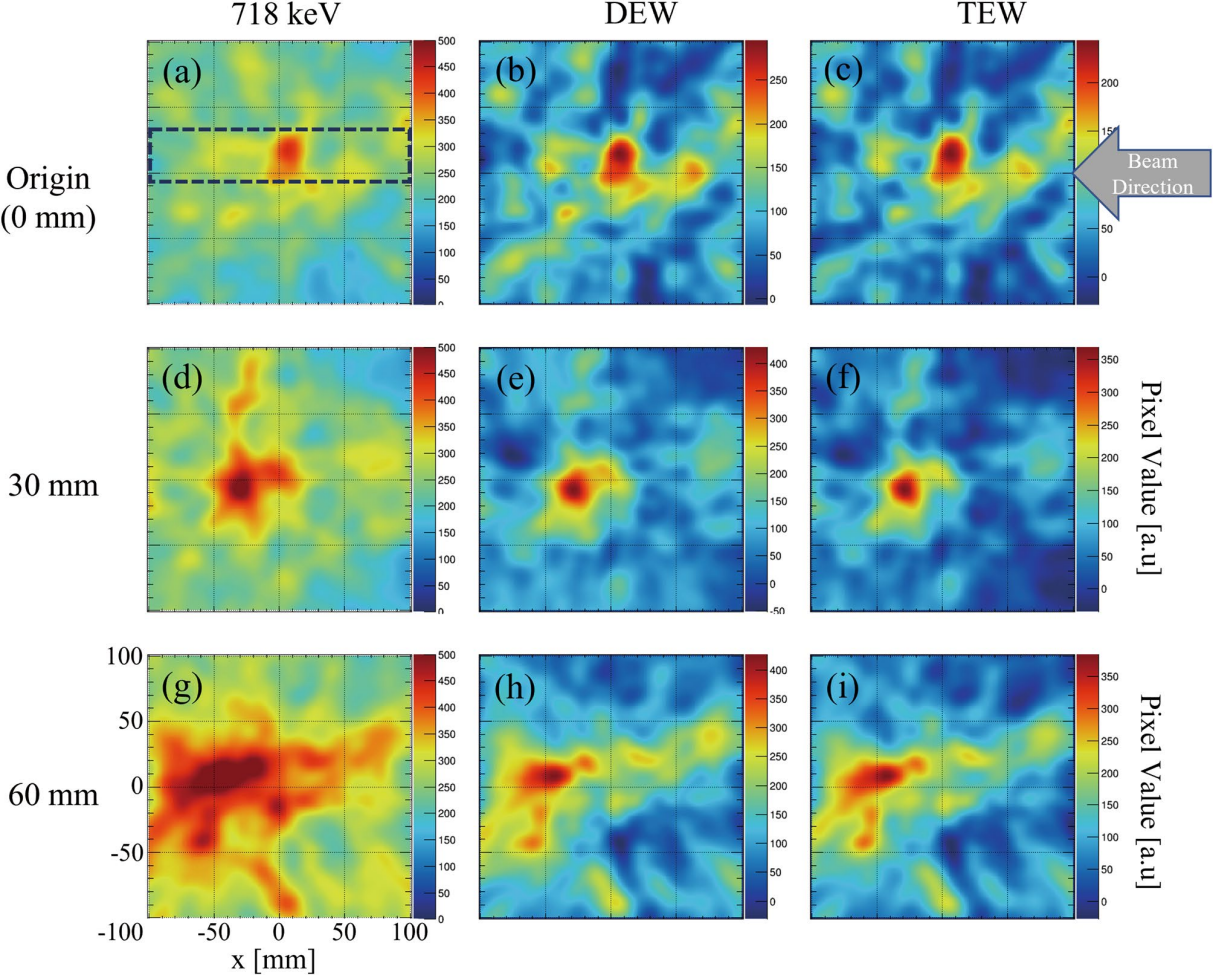
(**a**), (**d**), and (**g**) Reconstructed back-projection Compton images of 718 keV; (**b**), (**e**), and (**h**) the corresponding dual-energy window (DEW); (**c**), (**f**), and (**i**) and triple-energy window (TEW) images of the graphite phantoms positioned at origin (0 mm), 30 mm, and 60 mm, respectively. The dotted rectangular line in (**a**) indicates the projection range used to calculate x-projection profile of all the images in this figure. The arrow in (**c**) shows the direction and location of the beam entrance in graphite phantom.

We reconstructed the dual-energy window (DEW) and triple-energy window (TEW) images using the data with the energy window of 680 keV, 718 keV, and 760 keV to evaluate the effect of scattered gamma rays on the 718 keV prompt gamma images. Figure 5 shows the reconstructed images of 718 keV (Fig. 5a,d,g: same as Fig. 3b,e,h) and the corresponding DEW (Fig. 5b,e,h) and TEW (Fig. 5e,f,i) images for the phantom positioned at 0 mm, 30 mm, and 60 mm, respectively. The peak intensity positions in DEW images were located at − 1.0 mm, − 2.5 mm, and + 3.1 mm from the 718 keV prompt gamma images for the phantoms at 0 mm, 30 mm, and 60 mm, respectively. Furthermore, the peak intensity positions evaluated using TEW images were located at − 0.3 mm, − 1.5 mm, and + 5.9 mm from the 718 keV prompt gamma emission images for the phantoms at 0 mm, 30 mm, and 60 mm, respectively. Thus, the positions of the peak intensities of gamma emissions evaluated using DEW and TEW images were approximately at the same location as that of the imaging results with 718 keV alone.

## Discussion

We studied the feasibility of Compton camera for range verification of clinical carbon ion beam. The 718 keV prompt gamma rays emitted from the graphite phantom through nuclear reactions with projectiles were measured using a Si/CdTe Compton camera. The positions of peak intensities of the gamma emissions deviated from the BP positions by 4.0–13.3 mm. With the shift of the graphite phantoms by 30 mm and 60 mm, the peak intensities of gamma emission positions were also shifted. In this study, although the 60-mm shift was highly affected by statistical errors and angular positioning, the peak intensity positions of 718 keV prompt gammas in 0-mm and 30-mm phantom positions were closer to BP than those reported in previous studies of gamma measurements were^33,46^. It is unclear whether this deviation is because of the resolution of the Compton camera or the distribution of ^10^B*. Although the quantitative deviation is unknown at this time, the position of the BP should be related to the position where the most 718 keV gamma rays are generated. In fact, we confirmed that the peak position in the Compton image with 718 keV shifted in accordance with the position of the BP. Therefore, the Compton image with 718 keV could be used to estimate the BP position. To apply the range estimation, the imaging ability should be improved, and the accuracy confirmed. Concerning the angular resolution of our Compton camera with 5° for a 700 keV source kept 150 mm from the Compton camera detector, the spatial two-point resolution was 12.2 mm. If we visualize 718 keV gamma rays from the monoenergetic carbon beams and measure the peak position, we would be able to estimate BP with a higher resolution, although significant data is required. Considering the high dose concentration ability of CIRT, the spatial resolution of 1 cm is not good enough. However, in clinical practice the tumor may move several centimeters, which results in a significant dose reduction^47,48^. Though, further development of Compton camera would be necessary to achieve higher spatial resolution, the Compton images at this current state has potential to contribute to the confirmation of treatment effects and adaptive treatments.

Using a broad/wide beam of σ = 11 mm, we successfully reconstructed Compton images of 718 keV prompt gamma emissions. We expected an improved image quality for cases where small-sized beams such as those used in spot scanning techniques were used because the area of the high intensity position was small.

We verified the presence of 718 keV prompt gamma rays at the position of the Compton camera by using a cadmium zinc telluride (CZT) detector. The irradiated carbon ion beam specifications and the experimental setup were the same as those used in beam range monitoring with the Compton camera. The target phantom was cuboid polymethyl methacrylate (PMMA). The energy spectrum measured by the CZT detector clearly illustrated the presence of 718 keV prompt gamma rays at the position of the Compton camera placed for beam range monitoring (Supplementary Figure). This confirms that the 718 keV peak would be hidden among the background noise and would not be visible in the energy spectrum detected using the Compton camera. However, no traces of peak intensities of prompt gamma emissions were observed in the individual reconstructed images of 680 keV (Fig. 3a,d,g) and 760 keV (Fig. 3c,f,i), similar to that experienced in our previous studies^43^. This indicates that the peak intensities in the 718 keV images arise from the prompt gamma emissions (not from any scattered gamma rays from high-energy secondary radiations). The effects of shifts in phantom positions on peak intensity positions were also clearly observed in DEW and TEW images (Fig. 5); the resultant peak intensity positions were consistent with those of 718 keV prompt gamma images. The image contrast was also significantly improved in the DEW and TEW images because of the suppression of the effect of scattered radiation. At the same time, the pixel values in the surrounding area were reduced to approximately zero. Therefore, we expect that the TEW and DEW methods may help quantify carbon beam intensity.

A preliminary calculation of Monte Carlo simulations had shown that the generation of 10B* fragments responsible for 718 keV prompt gamma emissions evaluated for water and PMMA phantom was slightly less (20% and 10%, respectively) than in graphite phantom. Moreover, our preliminary results on the PMMA phantom did not show a significant difference from that obtained in the graphite phantom. However, due to the complex chemical composition of a human body, 718 keV generation may slightly be lower than the graphite phantom. In addition, we also set the thickness of the phantom to 15 mm (relatively very low value with respect to human size or human size phantom) to overcome the influence of scattering and reliably capture the prompt gamma. For practical use, it will be necessary to increase the detection efficiency significantly.

The Compton camera used in this study (ASTROCAM) is a commercial product developed for the purpose of environmental measurement. ASTROCAM has a high-energy resolution, but there are some parts that are not suitable for beam monitoring. We expect that improvement in terms of optimization of detector arrangement, energy thresholds, and application-specific integrated circuits (ASIC) would deliver better results. For example, ASTROCAM is equipped with a large number of sub-detectors, but in practice, only the pair of the last layer of Si and the first layer of CdTe acquired almost all data. Moreover, the other (less useful) sub-detectors cause accidental coincidence measurements (e.g., 3-hit events), which increase dead time and reduce the signal. The efficiency of the Compton camera could be improved by aligning the detectors at the side. The size of the current sub-detectors is 50 × 50 mm^2^. Considering the size of single-photon emission computed tomography (SPECT) and flat-panel detector, the efficiency could be increased 100 times or more. If we could establish a precise synchronization between the beam pulses and the Compton camera operation to measure prompt gamma rays generated during the “on” stage of the pulses, the ratio of the signal could be improved and noise during the “off” stage eliminated. If we can reduce the analog to digital conversion time by improving the ASIC, we would reduce the dead time by a factor of 10. We expect that improvements in the efficiency of the Compton camera by four or more orders of magnitude would be acceptable for the passive irradiation technique for efficient beam range monitoring. However, for the spot scanning irradiation technique, the efficiency of the Compton camera should be improved by five orders of magnitude. This can be accomplished by performing electron tracking techniques in the Si detector, depth analysis inside the CdTe detector, and improvements in image reconstruction algorithms.

The range of carbon ion beam can be imaged using 718 keV prompt gamma rays. Nevertheless, the current state of Compton camera is inefficient to directly adopt this system for clinical application. However, the results of this study reveal the feasibility of Compton camera for beam range verification, especially for 718 keV prompt gammas.

## Methods

### Compton camera

In this study, we used the Si/CdTe semiconductor-based Compton camera (ASTROCAM 7000HS; Mitsubishi Heavy Industries Ltd., Japan)^49^. Figure 1a shows the schematic illustration of the Compton camera used in this study. It consists of eight layers of Si detectors and four layers of CdTe detectors. The length and width of each of the detectors were 50 mm. The energy resolution of the Compton camera was 2.2% with respect to 662 keV gamma rays at full width at half maximum (FWHM). The angular resolution measure was 5.4° (FWHM) for 662 keV.

### Imaging method

Compton images in this study were reconstructed using the simple back-projection (uniformly enlarged projection) method^35,50^. We selected only the “two-hit events” (one hit in the Si detector and another in the CdTe detector) as a Compton event for image reconstruction. The energy window was set to 708–728 keV, and the threshold energy to determine a hit in both the detectors was set to 20 keV. The size of the field of view was 200 × 200 mm^2^, and the pixel size was 1 mm/px.

In some cases, the high-energy gamma rays simultaneously scatter in both the scatterer and absorber, and occasionally, we observe second scattering events of low-energy gamma rays in the absorber. The errant low-energy gamma rays generate artifacts in the reconstructed image. Conventionally, these artifacts are removed in SPECT by adopting DEW or TEW imaging methods^43^. In this study, we adopted DEW and TEW methods to evaluate the presence of crosstalk effects in the reconstructed 718 keV prompt gamma image from the surrounding energy windows. To reconstruct the DEW images, an upper-side energy window of 750–770 keV for 760 keV was considered, whereas, for TEW images, a lower-side energy window of 670–690 keV for 680 keV and an upper-side energy window of 750–770 keV for 760 keV were used.

### Heavy-ion carbon beam

All beam monitoring experiments were conducted using carbon beams at Gunma University Heavy Ion Medical Center (GHMC), Japan^51^. GHMC uses the passive irradiation technique for clinical treatments. In this study, the beam used was also passive irradiation without wobbling instead of spot scanning irradiation. A horizontal carbon ion beam with a Gaussian distribution (σ = 11 mm) was delivered to the target graphite phantom for 10 min each in all experiments. The monoenergetic beam energy and beam intensity were set at 290 MeV/u and 3 × 10^9^ particles per pulse in 3 s (i.e., 6 × 10^11^ carbon ions), respectively. The duration of the pulses was 3 s, comprising 1 s “on” and 2 s “off” pulses. The beam flux intensity was set to 1 × 10^9^ per second to represent an actual clinical situation of beam delivery (“clinical” in this study was a matter of flux instead of fluence), despite the chances of large noise generations, accidental coincidences, and longer dead time ratios. In the entire beam monitoring experiment, beam delivery and operation of the Compton camera began and ended simultaneously. The BP position estimated by Monte Carlo simulation using Particle and Heavy-Ion Transport code System^52^ under the experimental conditions was at 98.4 mm from the beam entrance position of the graphite phantom (Table 1). All the values of BP positions mentioned in this study was the position of the highest dose delivered to the target phantom in the MC simulation calculation. The attenuation of the beam energy due to air transmission from the nozzle to the target phantom was also considered in the Monte Carlo simulation.

### Experimental setup

Graphite phantoms (200 × 200 × 15 mm^3^, 1.71 g/cm^3^) were used. Figure 2a shows a schematic illustration of the experimental setup for on-beam imaging of prompt gamma emission. The precise alignment of the graphite phantom with the beam direction was confirmed using a three-dimensional laser level installed inside the experimental room. The total distance from the beam axis to the first layer of the Si detector plane in the Compton camera was 140 mm. First, the measurements were made with the graphite phantom placed at the origin (0 mm). (The center of the camera and the calculated peak position were aligned.) Subsequently, the phantom was replaced with a new one, and the installation positions were shifted 30 mm and 60 mm away from the origin in the beam direction. The shift of the graphite phantom was in a perpendicular direction to the Compton camera, as illustrated in Fig. 2a.

## Supplementary Information

Supplementary Figure 1.
