# Supplementary figures and images for: Carbon range verification with 718 keV Compton imaging
Source: Sci Rep. 2021 Nov 4;11:21696. doi: 10.1038/s41598-021-00949-5 (PMC8569035; doi:10.1038/s41598-021-00949-5)

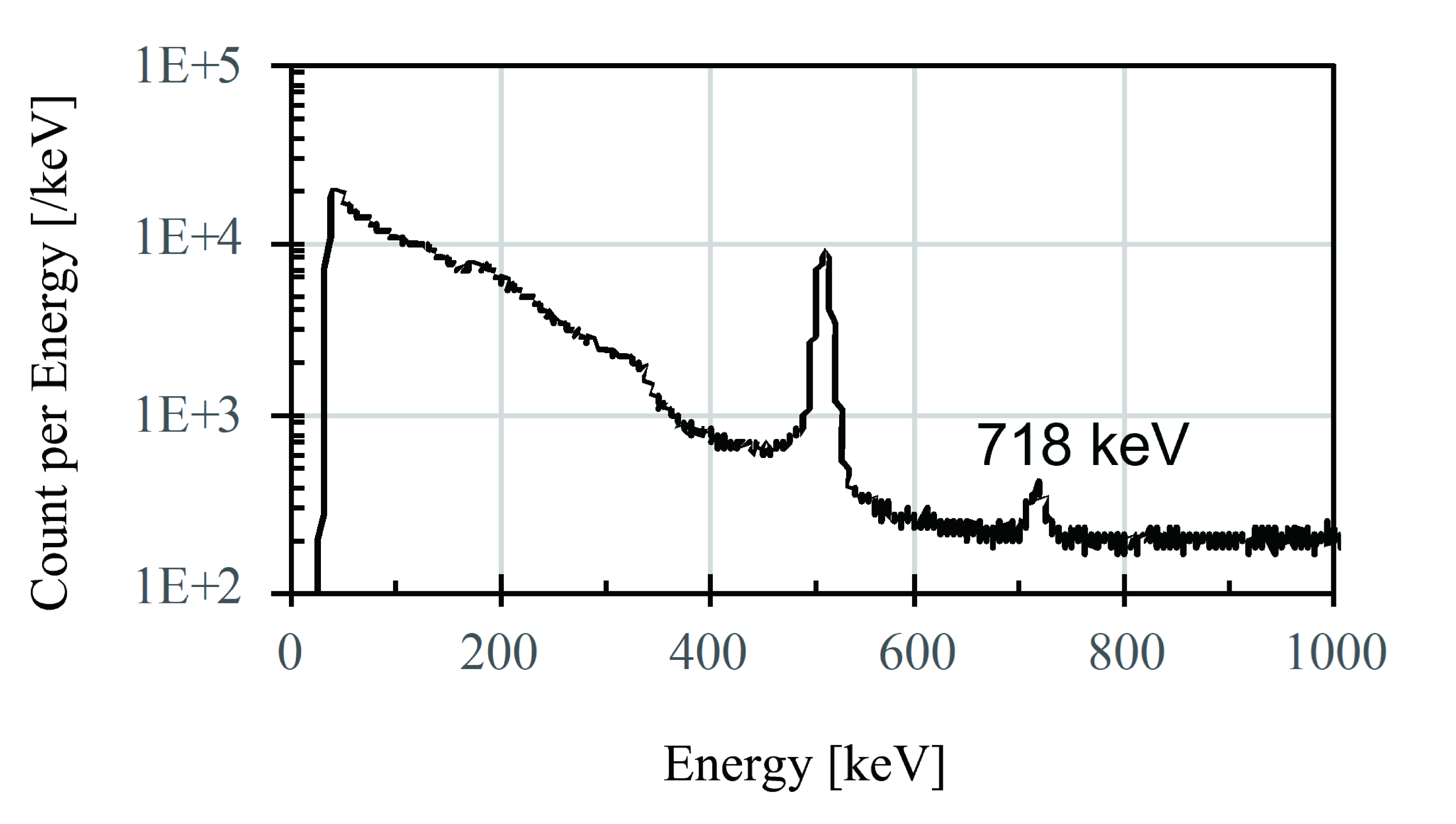

Supplement: Supplementary file 1 — Supplementary Figure 1. [file 41598_2021_949_MOESM1_ESM.tif]
